# The effect of exercise snacks on cardiorespiratory fitness, body composition, metabolic health, and muscle strength in sedentary individuals: a systematic review and meta-analysis

**DOI:** 10.3389/fnut.2026.1883923

**Published:** 2026-09-28

**Authors:** Kai Qi, Qiyi Wang, Decheng Xu, Adam Kawczyński, Aiguo Chen

**Affiliations:** 1 School of Sport and Brain Health, Nanjing Sport Institute, Nanjing, China; 2 Gdansk University of Physical Education and Sport, Gdansk, Poland; 3 College of Physical Education, Yangzhou University, Yangzhou, China; 4 Faculty of Medicine, Wrocław University of Science and Technology, Wrocław, Poland

**Keywords:** body composition, cardiorespiratory fitness, exercise snacks, metabolic health, muscle strength, sedentary

## Abstract

**Objective:**

This systematic review and meta-analysis aimed to investigate the effects of exercise snacks (ES) on cardiorespiratory fitness, body composition, metabolic health, and muscle strength in sedentary individuals.

**Methods:**

From database inception to February 5, 2026, four international databases (PubMed, Web of Science, SciELO, and the Cochrane Central Register of Controlled Trials) and one Chinese database (China National Knowledge Infrastructure) were searched. The GRADE approach was used to assess the certainty of evidence, while the Cochrane Risk of Bias tools (RoB 2) and Risk of Bias In Non-randomized Studies of Intervention (ROBINS-I) were applied to evaluate the quality of randomized controlled trials and non-randomized studies, respectively. The pooled effects of study outcomes were estimated using a random-effects model based on meta-analysis. Subgroup analyses and meta-regression were conducted to explore potential moderating variables.

**Results:**

A total of 22 studies involving 994 participants were included. The meta-analysis showed that ES significantly improved VO_2max_ (Hedges’ g = 1.24, 95% CI: 0.53 to 1.95, *p* < 0.001, I^2^ = 75.0%, τ^2^ = 1.13), VO_2peak_ (Hedges’ g = 0.63, 95% CI: 0.05 to 1.22, *p* < 0.05, I^2^ = 55.0%, τ^2^ = 0.19), triglycerides (Hedges’ g = 1.05, 95% CI: 0.05 to 2.06, *p* < 0.05, I^2^ = 84.0%, τ^2^ = 0.65), and blood glucose (Hedges’ g = −0.83, 95% CI: −1.51 to −0.15, *p* < 0.05, I^2^ = 78.0%, τ^2^ = 0.69) in sedentary populations. However, significant publication bias was detected for VO_2max_, and the trim-and-fill analysis yielded an adjusted effect estimate that was no longer statistically significant (Hedges’ g = 0.62, 95% CI: −0.59 to 1.83, *p* = 0.32). No significant differences were observed in body fat, muscle mass, strength, or insulin levels.

**Conclusion:**

As a short-duration, high-frequency intervention, ES may improve cardiorespiratory fitness and certain metabolic health indicators in sedentary individuals. However, the finding for VO_2max_ should be interpreted cautiously because the trim-and-fill adjusted estimate was not statistically significant, suggesting that publication bias may have influenced the observed effect. It provides a potentially sustainable and effective exercise intervention strategy for sedentary individuals.

**Systematic review registration:**

PROSPERO (CRD420261347411).

## 1 Introduction

With the rapid development of socioeconomics and technology, physical activity in daily life has markedly decreased, and a sedentary lifestyle has gradually become the norm, which is regarded as an independent health risk factor affecting populations worldwide (1). A sedentary lifestyle is closely associated with the occurrence of various chronic diseases, particularly declines in cardiopulmonary function (2), increases in metabolic disorders (3), rising obesity rates (4), and health problems such as muscle atrophy (5). In addition, a meta-analysis involving more than one million participants indicated that prolonged sedentary time increases the risk of mortality (6). These issues not only exert long-term adverse effects on individual health but also pose significant challenges to global public health. In its 2020 guidelines on physical activity, the World Health Organization (WHO) explicitly recommends that adults engage in at least 75 min of vigorous-intensity or 150 min of moderate-intensity aerobic exercise per week, supplemented by two or more sessions of strength training (7). Although the positive effects of regular physical activity on health have been extensively validated, including improvements in cardiovascular health (8, 9), enhanced muscle strength (10), optimized metabolic function (11), and weight control (12), many individuals still find it difficult to meet these standards due to time constraints, underlying medical conditions, and lack of appropriate exercise facilities (13, 14). To address this issue, an increasing number of studies have focused on improving sedentary behavior and exploring effective intervention strategies.

The term “exercise snacks (ES)” was first proposed by Hartley (15) in a 2007 article published in Newsweek. Exercise snacks align with the guidelines on physical activity and sedentary behavior issued by the World Health Organization (7), emphasizing the potential health benefits of short bouts of physical activity. ES is defined as short, efficient bouts of exercise distributed throughout the day, and as a convenient and effective strategy, it has been increasingly advocated to enhance physical activity levels, reduce sedentary behavior, and promote overall health and well-being (16, 17). This form of short-duration exercise not only helps sedentary individuals break prolonged periods of inactivity and reduce health risks associated with sedentary behavior, but also improves cardiorespiratory fitness, enhances muscle strength, and optimizes metabolic function without requiring substantial time commitment (18–20). Moreover, due to its flexibility and simplicity, ES is particularly suited to modern busy lifestyles, providing a feasible form of exercise for a broader population (21). Recent studies have shown that ES not only helps increase daily physical activity levels but also has positive effects on weight management and the prevention of chronic diseases, gradually becoming a key focus in the field of public health (22, 23).

Currently, ES has been increasingly applied among sedentary individuals, including activities such as stair climbing, bodyweight exercises, and walking (24–26). Although these interventions have yielded some positive outcomes, most existing studies are characterized by small sample sizes and short-term intervention trials, and the findings across studies remain inconsistent, lacking systematic and comprehensive synthesis. Therefore, there is an urgent need for more rigorous and comprehensive systematic reviews and meta-analyses to evaluate the long-term health effects of ES in sedentary individuals. This study will focus on the effects of ES on key health indicators, including cardiorespiratory fitness, body composition, metabolic health, and muscle strength, thereby providing a more authoritative evidence base for this field.

Based on this, the present study aims to systematically evaluate and synthesize the existing literature through a systematic review and meta-analysis to assess the effects of ES on cardiorespiratory fitness, body composition, metabolic health, and muscle strength in sedentary individuals. We will focus on analyzing the effects of ES on the following key outcomes: cardiorespiratory fitness (V̇O₂_peak_ and VO₂_max_), body composition (body fat and muscle mass), metabolic health (blood glucose, insulin, and triglyceride levels), as well as muscle strength. Through this study, we expect to provide scientific evidence for the application of ES and offer theoretical support for the development of health intervention strategies for sedentary individuals, thereby further promoting the implementation of effective interventions targeting sedentary behavior in the field of public health.

## 2 Materials and methods

This systematic review and meta-analysis was registered with PROSPERO (CRD420261347411) and conducted in accordance with the PRISMA guidelines and the Cochrane Handbook for Systematic Reviews of Interventions on 21 March 2026 (27).

### 2.1 Search strategy

From the establishment of the database until February 5, 2026, we conducted a comprehensive literature search across five major electronic databases: PubMed, Web of Science, SciELO, Cochrane Central Register of Controlled Trials (CENTRAL), and China National Knowledge Infrastructure (CNKI). The search strategy employed a combination of keywords and MeSH (Medical Subject Headings) terms to ensure the comprehensiveness and accuracy of the search. The detailed search strategy is provided in the Supplementary materials. For English literature, the search scope was limited to full-text articles published in English and involving human subjects, to ensure the broad applicability and relevance of the research. For Chinese literature, we conducted an additional search through CNKI (China National Knowledge Infrastructure), specifically looking for Chinese-language articles to avoid missing relevant Chinese studies.

To ensure the inclusion of all relevant studies, we also screened the reference lists of the articles retrieved by the filtering system to identify and further include additional relevant studies. Additionally, we manually searched relevant journals and conference papers to further supplement and verify the search results. All included studies underwent a rigorous screening process to ensure they met the study’s standards and quality requirements. Our search strategy included both English and Chinese literature but only incorporated studies involving human subjects to ensure the broad applicability of the research findings.

### 2.2 Eligibility criteria and study selection

A total of 22 studies were included, and the detailed study selection process is shown in Figure 1, which includes the detailed steps for identifying and including relevant studies. Duplicate references were removed using EndNote, and the two authors (KQ and QYW) screened the titles and abstracts of identified studies to determine which studies met the inclusion criteria. Subsequently, full texts of potentially eligible studies were reviewed to determine the final included studies. In case of any disagreement between the two authors, the third author (DCX) mediated and resolved the issue to determine the eligibility for inclusion. The selection of studies followed the PICOS criteria (28), with the specific criteria as follows: (i) Population: The included participants were sedentary adults aged 18 and above, typically not engaged in regular physical activity. (ii) Intervention: The experimental group received an exercise snack (ES) intervention. For the purposes of this review, ES was operationally defined as brief, structured, and time-efficient bouts or sessions of exercise, performed as an alternative to prolonged continuous exercise. Eligible interventions consisted of short periods of exercise performed daily, either as repeated bouts distributed throughout the day or as short-duration exercise sessions, with the aim of increasing physical activity levels. (iii) Comparator: The control group maintained their usual lifestyle or received a non-exercise intervention. Where stretching was used as a control intervention, it was intended to reduce muscle tension and improve flexibility rather than to enhance cardiorespiratory fitness or other physical activity-related outcomes. (iv) Outcomes: The main outcome measures included cardiorespiratory fitness (V̇O_2peak_, VO_2max_), body composition (body fat, muscle mass), strength, and metabolic health (blood glucose, insulin, triglycerides). (v) Study Design: The included studies were controlled trials, including randomized controlled trials (RCTs) and non-randomized controlled trials (n-RCTs).

**Figure 1:**
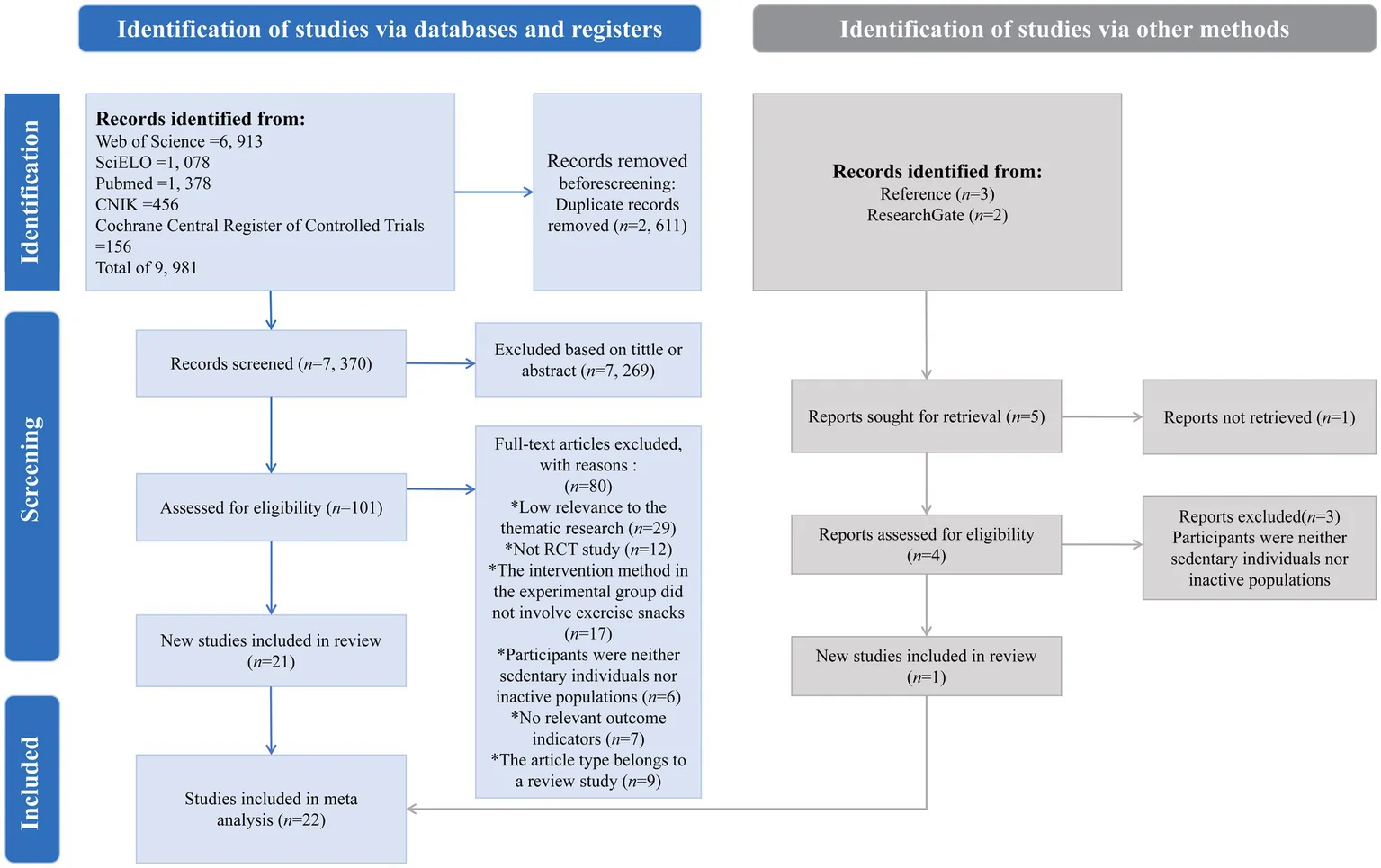
PRISMA flow diagram for included and excluded studies. CNKI, Chinese National Knowledge Infrastructure.

### 2.3 Data extraction

The data extraction for this study followed the guidelines outlined in the Cochrane Collaboration Handbook (29). After obtaining the final dataset, two researchers (KQ and QW) independently extracted data from the included studies and entered it into a spreadsheet. The spreadsheet was pilot-tested on a subset of the research samples to ensure clarity and consistency in the data extraction. Any discrepancies were resolved through consultation with a third researcher (DX). The extracted data included: (i) study details, such as the first author, year of publication, and country. (ii) Demographic information, including the number of participants, age, and gender. (iii) Participant characteristics, all of whom were sedentary or inactive individuals. (iv) Intervention characteristics, including the intervention duration, number of interventions per day, and frequency of intervention. (v) Outcome measures, methods of assessment, and key findings. Additionally, forest plots were generated for the meta-analysis using the mean change and standard deviation (SD) or mean differences and their corresponding outcome SDs. If the original studies did not report relevant data, efforts were made to contact the authors for additional information or use WebPlot Digitizer version 4.4^1^ to extract values from published graphs. Furthermore, the standard deviation (SD) of the mean change in this study was calculated as follows (30):

$$SD_{\text{change}}=\sqrt{SD_{\mathrm{pre}}^{2}+SD_{\text{post}}^{2}-(2\times r\times SD_{\mathrm{pre}}\times SD_{\text{post}})}$$

SD_change_ represents the standard deviation of the mean change, SD_pre_ represents the standard deviation before the intervention, SD_post_ represents the standard deviation after the intervention, and r is the correlation coefficient (30). Since very few studies report the correlation coefficient r for pre- and post-comparison. According to the recommendations of the Cochrane Handbook, the value of r is typically assumed to be 0.50.

### 2.4 Risk of Bias assessment

For the included RCTs, the Cochrane Risk of Bias 2 (RoB 2) tool was used for assessment (31), with studies classified as “low risk,” “some concerns,” or “high risk” of bias. The assessment covered five domains: bias arising from the randomization process, bias due to deviations from intended interventions, bias due to missing outcome data, bias in measurement of the outcome, and bias in selection of the reported result. For the included N-RCTs, the Risk of Bias In Non-randomized Studies of Intervention (ROBINS-I) tool was used for assessment, with studies classified as “low risk,” “moderate risk,” “serious risk,” or “critical risk” (32). Each included study was jointly assessed by two researchers (KQ and QW), and any discrepancies in any domain were resolved through discussion, if possible; if consensus could not be reached, a third researcher (DX) was consulted to achieve consensus.

### 2.5 Certainty of evidence

This study used the GRADE (Grading of Recommendations, Assessment, Development, and Evaluation) framework to assess the certainty of evidence for each outcome measure (33). According to the GRADE criteria, the certainty of evidence is classified into four levels: high, moderate, low, and very low. The assessment of certainty of evidence takes into account factors such as study design, risk of bias, consistency of results, sample size, missing data, and publication bias. In evidence classification, high-quality evidence indicates rigorous study design, consistent results, and reliable conclusions; moderate-quality evidence suggests certain limitations in the study, but the results are still credible; low-quality evidence implies the presence of bias or heterogeneity, and the conclusions are more uncertain; very low-quality evidence indicates serious methodological flaws, making the conclusions unreliable (34). By applying the GRADE framework, we conducted a rigorous assessment of the certainty of evidence for all outcomes, providing stronger support for the final conclusions.

### 2.6 Statistical analysis

The statistical analysis in this study was performed using R v 4.4.2 software (35). For continuous outcome measures, the Hedges’ g and its 95% confidence interval (CI) were used as effect size indices for the combined analysis. Given the potential clinical and methodological differences among the included studies, a random-effects model was prioritized for the meta-analysis to obtain a more conservative and extrapolatable combined effect size. Between-study heterogeneity was assessed using Cochran’s Q test and the *I*^2^ statistic, with the *I*^2^ value used to quantify the degree of heterogeneity. When I^2^ ≤ 50%, heterogeneity is considered low; when I^2^ > 50%, moderate to high heterogeneity is considered present. In addition, to explore the sources of heterogeneity and potential moderators, this study employed subgroup analysis and meta-regression, incorporating both categorical and continuous variables (36). It is generally recommended that meta-regression analysis include at least 10 studies (37, 38). To further explore potential sources of heterogeneity, sensitivity analysis was performed by excluding each study one at a time (leave-one-out method) to assess the impact of individual studies on the overall combined results and inter-study heterogeneity, thus testing the robustness of the results. When the meta-analysis includes at least 10 studies, a funnel plot is used for visual assessment of publication bias, and Egger’s linear regression test is used to investigate asymmetry in the funnel plot. All statistical tests were two-tailed, with a significance level set at *p* < 0.05.

In addition, when a single publication contributed data from multiple eligible experimental groups or independent comparisons, each independent comparison was included separately in the meta-analysis, whereas the publication was cited only once in the text.

## 3 Results

### 3.1 Study selection

This study used a PRISMA flowchart to document the study selection process (Figure 1). Initially, 9,981 articles were screened from four English-language databases and one Chinese database, with 2,611 duplicate articles removed, leaving 7,370 articles. Subsequently, after screening titles and abstracts, 101 full-text articles were further assessed, and 80 records were excluded for the following reasons: (i) low relevance to the topic (*n* = 29), (ii) not a controlled trial (*n* = 12), (iii) the intervention in the experimental group was not classified as exercise snacks (*n* = 17), (iv) participants were not from sedentary or inactive populations (*n* = 6), (v) no relevant outcome measures (*n* = 7), (vi) the article was a review (*n* = 9). One study was included from other sources. Ultimately, 22 studies met the inclusion criteria for the meta-analysis.

### 3.2 Characteristics of included studies

This study includes 22 studies (18, 22, 24, 39–57), and the participant characteristics and intervention protocols of each study are presented in Tables 1, 2. The participants were all sedentary and inactive individuals, with a total of 994 participants. The duration of interventions varied significantly, ranging from 1 day to 12 weeks, which may affect the variability of the results. Additionally, the types of ES varied across studies: 8 studies used stair climbing (22, 39, 44, 45, 47, 48, 53, 56), 2 studies used strength training (41), 2 studies used sprint training (43, 57), 2 studies used brisk walking (42, 50), 4 studies used different intensities of interval training (45, 47, 51, 52), 1 study used traditional exercise programs (40), 1 study used lifestyle-based exercise snacks (54), and 2 studies did not specify the type of exercise snack (18, 55).

**Table 1:** Basic information from Characteristic of participants.

Study	Country	Design	Participants	Sample size(n)		Sex	Outcome indicators	Measuring tools	Outcomes presented in the study
				Experimental; Age (years)	Control; Age (years)				
Zhou et al. (2025) (39)	China	RCT	Sedentary obese adults	14; (22.14 ± 1.88)	13; (21.08 ± 1.32)	Male, Female	Body Composition	DEXA	The weight, fat percentage, total fat mass, android, and trunk area fat mass in the snack group substantially improved.
Colvin et al. (2024) (40)	UK	RCT	Sedentary adults	A: 5; (26 ± 2.5) B: 5; (26 ± 3.25)	5; (26 ± 2.5)	Male, Female	Glycemic control	Glucometer	Three minutes of yoga can improve blood sugar in Sedentary adults, but tai chi cannot.
Jenkins et al. (2019) (22)	Canada	RCT	Sedentary adults	12; (20.0 ± 1.8)	12; (19.3 ± 1.6)	Male, Female	Cardiorespiratory fitness	HRM, RPES	The stair climbing “snacks” are effective in improving cardiorespiratory fitness
Babir et al. (2020) (24)	Canada	RCT	Sedentary adults	33; (53 ± 6)	38; (54 ± 7)	Male, Female	Aerobic capacity, Blood biomarkers, SPS, EE	LES, RPE, EES, Blood Analysis	Twelve weeks of remotely delivered exercise snacks were feasible for inactive adults to complete.
Brandt et al. (2024) (41)	Germany	N-RCT	Sedentary women	15; (42.1 ± 11.1)	15; (49.9 ± 9.7)	Female	Muscle mass	SECA® 213, BackCheck® 617, Y-Balance test, 6-min walking test,	RES could be an effective approach for the WHP to promote RT in inactive women with sedentary occupations and improve their muscle mass.
Perkin et al. (2019) (18)	UK	N-RCT	Sedentary older adults	10; (70 ± 4)	10; (74 ± 5)	Male, Female	Muscle Function	Physical activity monitor	Exercise snacking might be a promising strategy to improve leg muscle function and size in older adults.
Mues et al. (2025) (42)	Germany	RCT	Sedentary middle-aged adults	12; (50.6 ± 5.6)	13; (52.2 ± 5.1)	Male, Female	Cognitive Performance	TMT, SCWT	That workplace-integrated brief, vigorous exercise may lead to both immediate and sustained enhancement in executive functions.
Gillen et al. (2016) (43)	Canada	N-RCT	Sedentary men	9; (27.0 ± 7.0)	6; (26.0 ± 8.0)	Male	Indices of Cardiometabolic Health	EBCG, Metabolic cart, EBE	Short-term high-intensity interval training can improve the cardiometabolic health indicators of sedentary men.
Michael et al. (2021) (44)	UK	RCT	Sedentary women	A: 26; (31.76 ± 1.33) B: 24; (31.58 ± 1.41)	10; (32.0 ± 2.75)	Female	Physical fitness, inflammatory markers	FAS, BPM, MSFT, CMJ, Cobas 400 plus Analyzer	Stair use at home offers a low-cost intervention for disease risk reduction to public health.
Reljic et al. (2020) (45)	Germany	RCT	Severely obese individuals	30; (48.5 ± 10.0)	19; (49.0 ± 9.9)	Male, Female	Cardiometabolic health, work ability	BPM, BS, ABCM, CET, ASRM	HIIT may induce significant improvements in cardiometabolic health, especially VO_2max_, WAI and well-being in obese individuals after only 12 weeks.
Boreham et al. (2005) (46)	UK	RCT	Sedentary women	8; (18.9 ± 0.6)	7; (18.7 ± 0.8)	Female	Cardiorespiratory fitness	CET	Stair climbing activity can favourably alter important cardiovascular risk factors in previously sedentary young women.
Metcalfe et al. (2012) (47)	UK	RCT	Sedentary adults	A: 7; (26 ± 3) B: 6; (19 ± 1)	A: 8; (4 ± 3) B: 8; (21 ± 1)	Male, Female	metabolic health	OGTT, GCT	REHIT may offer a genuinely time-efficient alternative to HIT for improving risk factors of T2D.
Boreham et al. (2000) (48)	UK	RCT	Sedentary women	12; (19.8 ± 0.3)	10; (20.3 ± 0.3)	Female	Cardiovascular health	Vitros 950 IRC automated analyzer	A short-term stair-climbing program canconfer considerable cardiovascular health benefits onpreviously sedentary young women.
Yin et al. (2024) (49)	China	RCT	Inactive Adults	14; (22.1 ± 2.1)	15; (20.9 ± 1.4)	Male, Female	Cardiorespiratory Fitness	BIAD, RET	Exercise Snacks are a Time-Efficient Alternative to Moderate-Intensity Continuous Training for Improving Cardiorespiratory Fitness in Inactive Adults
Rodriguez and Wadsworth (2019) (50)	Costa Rica	RCT	Sedentary office employees	A: 24; (46 ± 9) B: 22; (48 ± 9)	22; (42 ± 10)	Male, Female	Aerobic fitness, body composition	Submaximal Bruce Protocol, iDXA	Walking has a positive effect on the aerobic fitness and physical condition of office workers who sit for long periods.
Bouaziz et al. (2018) (51)	France	RCT	Sedentary seniors	30; (72.9 ± 2.5)	30; (74.3 ± 3.4)	Male, Female	Cognitive, mental health, functional performance and quality of life	VFT, TMT, PASAT, GADS, SF-12, 6-MWT, TUG, OLBT, SPPB, CPET	IATP-R is an effective training programme to improve functional and cognitive performances, mental health and well-being in sedentary seniors.
Liao et al. (2026) (52)	China	RCT	Sedentary women	54; (23.69 ± 2.78)	42; (23.38 ± 3.71)	Female	Balance, ankle function, and physical fitness	SOT, CAIT	An 8-week HICT program appears to effectively improve balance and ankle stability in sedentary young women with FAI.
Yun et al. (2023) (53)	Korea	N-RCT	Sedentary women	12; (23.5 ± 4.7)	13; (26.4 ± 3.9)	Female	Cardiorespiratory Fitness, body composition	SOCST, MCSS	Short bouts of vigorous stair climbing is a feasible and time-efficient exercise strategy for improving CRF in previously sedentary, young women with overweight and obesity.
Macfarlane et al. (2006) (54)	China	RCT	Sedentary adults	20; (49.8 ± 6.5)	25; (47.7 ± 7.2)	Male, Female	Aerobic fitness, body composition	ART, EC	Accumulating multiple short bouts of LMPA, of which ∼50% were ≤6 min, can provide significant improvements in the fitness of sedentary adults.
Fang et al. (2026) (55)	China	RCT	Sedentary office workers	40; (37.2 ± 7.8)	39; (38.4 ± 7.4)	Male, Female	Blood glucose, Insulin, Anthropometric measures	OGTT, calibrated portable, Tanita BC-418	Hourly 3-min micro-exercise breaks represent a feasible and effective strategy for improving metabolic health in sedentary office workers.
Andersen et al. (2013) (56)	UK	RCT	Sedentary office workers	106; (42 ± 10)	54; (43 ± 11)	Male, Female	Aerobic Fitness, body composition	CET	Stair-walks together with colleagues at work improves cardiovascular health among adults in sedentary occupations.
Songsorn et al. (2016) (57)	UK	RCT	Sedentary individuals	15; (24 ± 6)	15; (23 ± 5)	Male, Female	Cardiorespiratory Fitness	CET	Exercise training comprising of single 20-s cycle sprints does not provide a sufficient stimulus for improving maximal aerobic capacity in sedentary individuals

DEXA, Dual-energy X-ray absorptiometry; HRM, heart rate monitor; RPES, rating of perceived exertion scale; SPS, Subjective physical sensation; EE, Exercise Enjoyment; LES, Bicycle force gauge; RPE, rating of perceived exertion; EES, exercise enjoyment scale; TMT, Trail Making Test; SCWT, Stroop Color–Word Test; EBCG, Electronically-braked cycle ergometer; EBE, electronically-braked ergometer; FAS, FitBit Aria Scale; BPM, blood pressure monitor; MSFT, Multi-Stage Fitness Test; CMJ, counter movement jump; BS, Blood sampling; ABCM, Anthropometric and body composition measurements; CET, Cycle ergometer test; ASRM, Assessment of self-reported measures; GCT, graded cycling test; OGTT, Oral Glucose Tolerance Test; REHIT, Reduced-Exertion High-intensity interval training; BIAD, bioelectrical impedance analysis device; RET, ramp exercise test; A, Experimental Group 1; B, Experimental Group 2; iDXA, Integrated Dual-energy X-ray Absorptiometry; VFT, Verbal fluency tasks; TMT, Trail making test; PASAT, Paced auditory serial addition test; GADS, Goldberg anxiety and depression scale; SF-12, Quality of life assessment using the 12-item Short form Health Surveys; 6-MWT; The 6-min walk test; TUG, Timed Up and Go test; OLBT, One-leg balance test; SPPB, Short physical performance battery tests; CPET, The cardiopulmonary exercise testing; SOT, Sensory Organization Test; CAIT, Cumberland Ankle Instability Tool; SOCST, standard open-circuit spirometry technique; MCSS, mechanical column scale with stadiometer; POSS, portable, open-circuit spirometry system; ART, Astrand-Rhyming test; EC, electromagnetic cycle.

**Table 2:** Basic information from Intervention protocol.

Study	Groups	Mode	Time	Interval time	Frequency	Intensity	Cycle
Zhou et al. (2025) (39)	Experimental group	Stair climbing	6 time	60 min	4 sessions/week	N/A	12 weeks
	Control group	No-exercise	N/A	N/A	N/A	N/A	12 weeks
Colvin et al. (2024) (40)	Experimental group A	Yoga	6 time	30 min	6 sessions/week	Low-intensity	1 day
	Experimental group B	Tai Chi	6 time	30 min	6 sessions/week	Low-intensity	1 day
	Control group	No-exercise	N/A	30 min	N/A	N/A	1 day
Jenkins et al. (2019) (22)	Experimental group	Stair climbing	3 time	60 min	6 sessions/week	N/A	6 weeks
	Control group	No-exercise	N/A	N/A	N/A	N/A	6 weeks
Babir et al. (2020) (24)	Experimental group	bodyweight exercises	3 time	N/A	3 sessions/week	High-intensity	12 weeks
	Control group	stretching	N/A	N/A	3 sessions/week	N/A	12 weeks
Brandt et al. (2024) (41)	Experimental group	resistance exercise snacking	1 time	0	5 sessions/week	N/A	12 weeks
	Control group	No-exercise	N/A	N/A	N/A	N/A	12 weeks
Perkin et al. (2019) (18)	Experimental group	exercise snacking	2 time	N/A	7 sessions/week	N/A	1 weeks
	Control group	No-exercise	N/A	N/A	N/A	N/A	1 weeks
Mues et al. (2025) (42)	Experimental group	running on the spot	3 time	60 min	4 sessions/week	High-intensity	4 weeks
	Control group	No-exercise	N/A	N/A	N/A	N/A	4 weeks
Gillen et al. (2016) (43)	Experimental group	Sprint interval training	3 time	N/A	3 sessions/week	High-intensity	12 weeks
	Control group	No-exercise	N/A	N/A	N/A	N/A	12 weeks
Michael et al. (2021) (44)	Experimental group A	Home-Based Stair Climbing	2 time	10 min	5 sessions/week	N/A	8 weeks
	Experimental group B	Gym-Based Stair Climbing	2 time	10 min	5 sessions/week	N/A	8 weeks
	Control group	No-exercise	N/A	N/A	N/A	N/A	8 weeks
Reljic et al. (2020) (45)	Experimental group	High-intensity interval training	5 time	1 min	2 sessions/week	High-intensity	12 weeks
	Control group	No-exercise	N/A	N/A	N/A	N/A	12 weeks
Boreham et al. (2005) (46)	Experimental group	Stair climbing	3 time	60 min	2–5 sessions/week	N/A	12 weeks
	Control group	No-exercise	N/A	N/A	N/A	N/A	12 weeks
Metcalfe et al. (2012) (47)	Experimental group	REHIT	1–2 time	0–220 min	3 sessions/week	High-intensity	6 weeks
	Control group	No-exercise	N/A	N/A	N/A	N/A	6 weeks
Boreham et al. (2000) (48)	Experimental group	Stair climbing	1–6 time	N/A	5 sessions/week	N/A	7 weeks
	Control group	No-exercise	N/A	N/A	N/A	N/A	7 weeks
Yin et al. (2024) (49)	Experimental group	Stair climbing	3 times	60 min	3 sessions/week	Medium-intensity	6 weeks
	Control group	No-exercise	N/A	N/A	N/A	N/A	6 weeks
Rodriguez and Wadsworth (2019) (50)	Experimental group A	multiple bouts of walking	N/A	N/A	N/A	Medium-intensity	10 weeks
	Experimental group B	continuous walking	N/A	N/A	N/A	Medium-intensity	10 weeks
	Control group	No-exercise	N/A	N/A	N/A	N/A	10 weeks
Bouaziz et al. (2018) (51)	Experimental group	IATP-R	6 time	3 min	2 sessions/week	N/A	9.5 weeks
	Control group	No-exercise	N/A	N/A	N/A	N/A	9.5 weeks
Liao et al. (2026) (52)	Experimental group	HICT	1 time	3 min	3 sessions/week	High-intensity	8 weeks
	Control group	No-exercise	N/A	N/A	N/A	N/A	8 weeks
Yun et al. (2023) (53)	Experimental group	Stair climbing	3 time	2 min	5 sessions/week	High-intensity	4 weeks
	Control group	No-exercise	N/A	N/A	N/A	N/A	4 weeks
Macfarlane et al. (2006) (54)	Experimental group	Lifestyle	5 time	N/A	4–5 sessions/week	N/A	8 weeks
	Control group	Exercise Prescription Model	1 time	0	4–5 sessions/week	N/A	8 weeks
Fang et al. (2026) (55)	Experimental group	Micro-exercise breaks	7 time	3 min	N/A	Low-intensity	12 weeks
	Control group	No-exercise	N/A	N/A	N/A	N/A	12 weeks
Andersen et al. (2013) (56)	Experimental group	Stair climbing	1 time	0	5 sessions/week	N/A	10 weeks
	Control group	No-exercise	N/A	N/A	N/A	N/A	10 weeks
Songsorn et al. (2016) (57)	Experimental group	Wingate sprint	1 time	N/A	3 sessions/week	N/A	4 weeks
	Control group	No-exercise	N/A	N/A	N/A	N/A	4 weeks

REHIT, Reduced-Exertion High-intensity interval training; A, Experimental Group 1; B, Experimental Group 2; IATP-R, Interval aerobic training programme with active recovery bouts; HICT, high-intensity circuit training.

Among the 22 studies, 5 were from China (39, 49, 52, 54, 55), 8 were from the UK (18, 40, 44, 46–48, 56, 57), 3 were from Canada (22, 24, 43), 3 were from Germany (41, 42, 45), and 1 each was from South Korea (53), France (51), and Costa Rica (50). In terms of experimental design, 18 studies were randomized controlled trials (RCT) (22, 24, 39, 40, 42, 44–52, 54–57), and 4 studies were non-randomized controlled trials (N-RCT) (18, 41, 43, 53).

### 3.3 Primary analysis of the results

For each outcome, studies were allocated to the corresponding synthesis based on the predefined eligibility criteria and the availability of relevant outcome data. Therefore, the number and composition of studies varied across the eight outcome syntheses.

#### 3.3.1 Effects of ES on cardiorespiratory fitness

Regarding VO_2max_, a total of 11 studies provided relevant data (22, 44–47, 49, 54, 56, 57). Preliminary analysis results show that the ES group showed significant improvement in VO_2max_ compared to the control group (Hedges’ g = 1.24, 95% CI: 0.53 to 1.95, *p* < 0.001, I^2^ = 75.0%, τ^2^ = 1.13). This result indicates that the ES intervention has a significant positive effect on improving VO_2max_. However, the high I^2^ value suggests considerable heterogeneity between studies, which may be related to study design, participant characteristics, or specific details of exercise intervention implementation. Therefore, although the overall results indicate a strong intervention effect, caution is still needed in interpreting the findings.

Regarding VO_2peak_, 4 studies provided relevant data (22, 24, 43, 53). Preliminary analysis results show that the ES group also exhibited significant improvement in VO_2peak_ compared to the control group (Hedges’ g = 0.63, 95% CI: 0.05 to 1.22, *p* < 0.05, I^2^ = 55.0%, τ^2^ = 0.19). This result suggests that the ES intervention has a significant positive effect on VO_2peak_. However, the I^2^ value of 55.0% indicates moderate heterogeneity, and despite the overall significant effect, the potential impact of these heterogeneity factors on the results should be considered. Due to the limited number of studies included, caution is advised when interpreting the results, and further research is needed to validate this finding. Future studies should provide more evidence, particularly in terms of sample size and standardization of intervention design, to increase the robustness of the research and the reliability of the results.

#### 3.3.2 Effects of ES on body composition

Regarding muscle mass, five studies provided relevant data (18, 41, 52, 53). The preliminary analysis showed that the ES group did not show significant improvement in muscle mass compared to the control group (Hedges’ g = 0.19, 95% CI: −0.21 to 0.59, *p* = 0.36, I^2^ = 56.0%, τ^2^ = 0.11). Similarly, for body fat, 11 studies provided relevant data (18, 39, 41, 43–45, 50, 52, 53), and no significant improvement was observed (Hedges’ g = 0.47, 95% CI: −0.39 to 1.33, *p* = 0.29, I^2^ = 87.0%, τ^2^ = 1.94). This result suggests that although ES interventions show a trend of improvement in body composition, the current evidence is insufficient to draw definitive conclusions as the statistical analysis did not reach significance.

#### 3.3.3 Effects of ES on metabolic health

Regarding Insulin, the data from five studies (24, 43, 47, 55) did not show significant improvement. However, significant improvements were found in three studies on Triglycerides (Hedges’ g = 1.05, 95% CI: 0.05 to 2.06, *p* < 0.05, I^2^ = 84.0%, τ^2^ = 0.65) (44, 45) and eight studies on Blood glucose (Hedges’ g = −0.83, 95% CI: −1.51 to −0.15, *p* < 0.05, I^2^ = 78.0%, τ^2^ = 0.69) (40, 43–45, 47, 55). Specifically, the ES intervention showed relatively stable positive effects in the improvement of Triglycerides and Blood glucose. These results suggest that, although improvements in insulin did not reach statistical significance, the ES intervention may have a significant positive impact on other metabolic health indicators, particularly Triglycerides and Blood glucose. Future studies may further explore the potential effects of ES on insulin and assess the intervention effects across different populations to ensure the broad applicability of the results.

#### 3.3.4 Effects of ES on muscle strength

Regarding strength, four studies provided relevant data. Preliminary analysis results showed no significant improvement in strength in the ES group compared to the control group (Hedges’ g = 0.34, 95% CI: −0.62 to 1.31, *p* = 0.49, I^2^ = 85.0%, τ^2^ = 0.84). This result may be related to the small number of studies included and the large heterogeneity between the studies. The high I^2^ value indicates significant differences between the studies, which may be due to factors such as study design, intervention intensity, or sample characteristics. Therefore, although no significant intervention effect was observed on strength, given the small number of studies and large heterogeneity, the robustness of the results needs to be interpreted with caution. Future studies should further increase sample size and standardize intervention methods to better validate the potential effects of ES on strength.

Overall, existing evidence suggests that ES shows relatively stable positive effects in improving cardiorespiratory fitness (VO_2max_ and VO_2peak_) and metabolic health (triglycerides and blood glucose), but its effects on body composition and muscle strength are more limited, and due to high heterogeneity, caution is needed in interpretation (Figure 2). Considering that the intensity of ES is relatively light and mainly focuses on short-duration, intermittent exercise interventions, it may be insufficient to directly improve indicators such as body composition and strength in sedentary individuals. Therefore, future research should further explore more reliable empirical studies and provide more evidence to validate the effects of exercise snacks on various health outcomes, especially regarding the optimization of intervention design and standardization.

**Figure 2:**
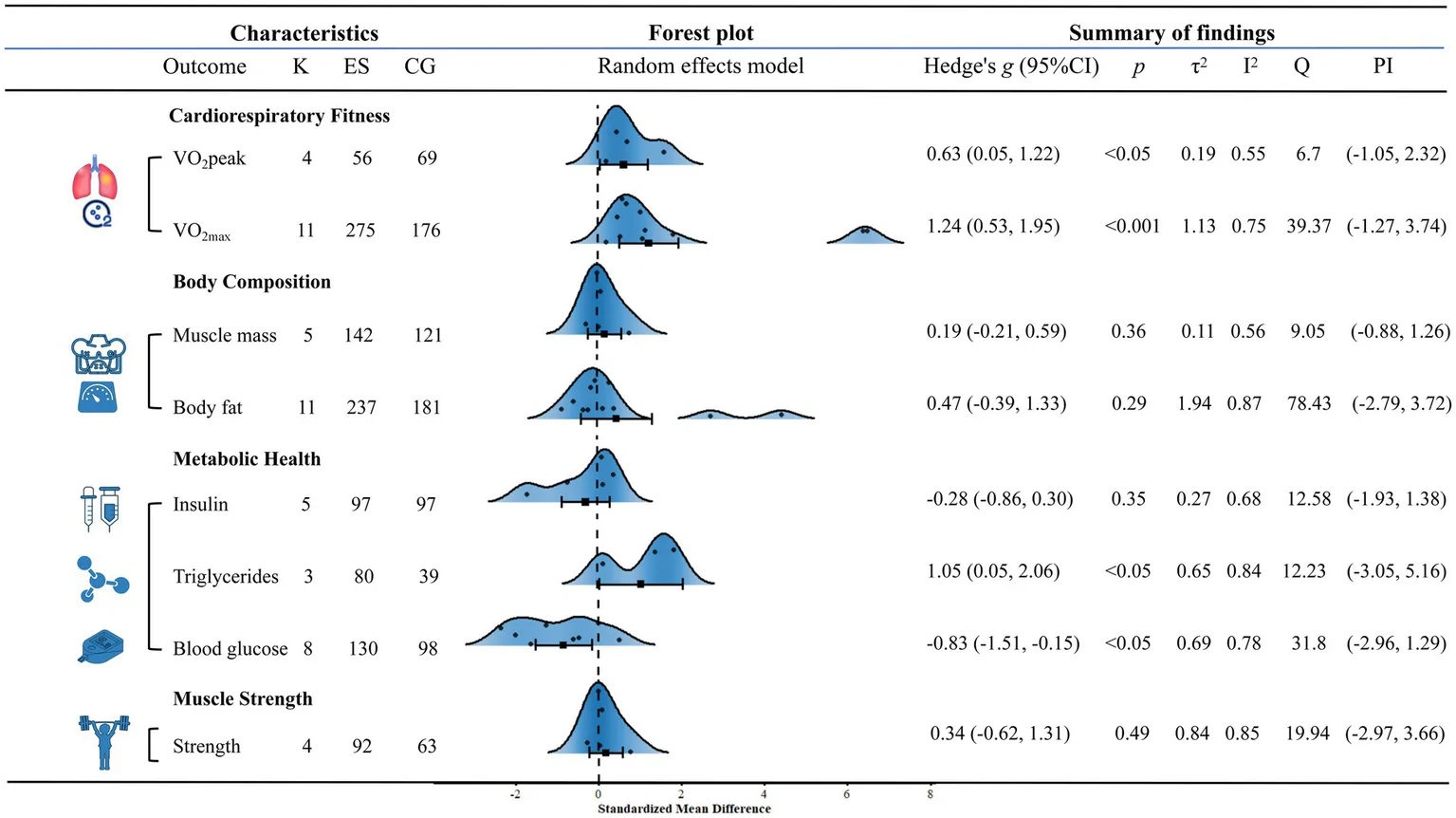
Pooled effect sizes for the outcomes. ES, exercise snacks; CG, control group; Hedge’ s g: the effect size indicators used in the pooled; τ^2^: represents the between-study variance, quantifying the extent of true heterogeneity; I^2^: quantitative indicators of heterogeneity; K: the total number of effects included in the pooled effect size; *p* value: statistically significant *p* values for pooled results; 95% CI = 95% confidence interval; Q: the null hypothesis that all studies share a common effect size; a significant Q (*p* < 0.05) indicates substantial heterogeneity; PI: provides the range within which the true effect size of a future similar study would fall, given the observed distribution of effects.

### 3.4 Subgroup analysis

We conducted a subgroup analysis to explore the moderating effects of participant gender, intervention frequency, intervention duration, and age on VO_2max_ (Figure 3) (Supplementary Table 1).

**Figure 3:**
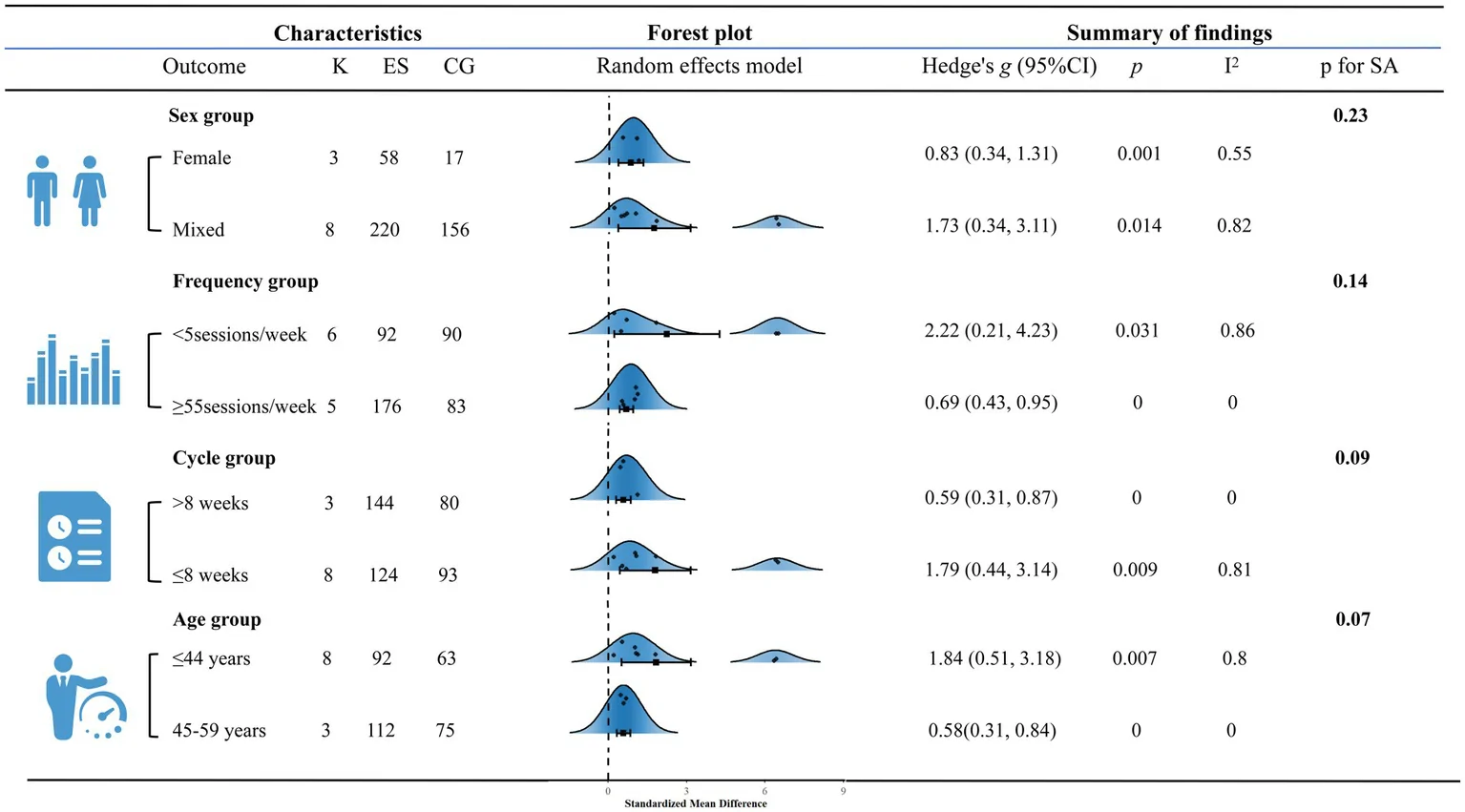
Subgroup analyses based on VO_2max_. ES: exercise snacks; CG: control group; Hedge’s g: the effect size indicators used in the pooled; I^2^: quantitative indicators of heterogeneity; *p* value: statistically significant *p* values for pooled results; 95% CI, 95% confidence interval.

### 3.5 Risk of bias in studies

We used the RoB 2 tool and ROBINS-I tool to assess the risk of bias in the included randomized controlled trials (RCTs) and non-randomized controlled trials (N-RCTs), with the specific results shown in Figure 4. The assessment results indicate that, among the 18 included RCT studies, there are concerns about biases arising from the randomization process, measurement of outcomes, and selective reporting, with some studies showing a high risk of bias. In the included N-RCT studies, about 50% of the studies showed a high risk of bias, and 25% displayed bias issues due to confounding factors. Overall, both RCT and N-RCT studies exhibit a certain level of bias risk across multiple dimensions, including some with more serious bias risks. It is recommended to combine funnel plots and Egger’ s test for further interpretation in subsequent analyses to better assess the robustness of the studies and clarify potential sources of bias. This analysis provides the necessary basis for the interpretation of subsequent results and reminds us to maintain a cautious attitude towards the research findings.

**Figure 4:**
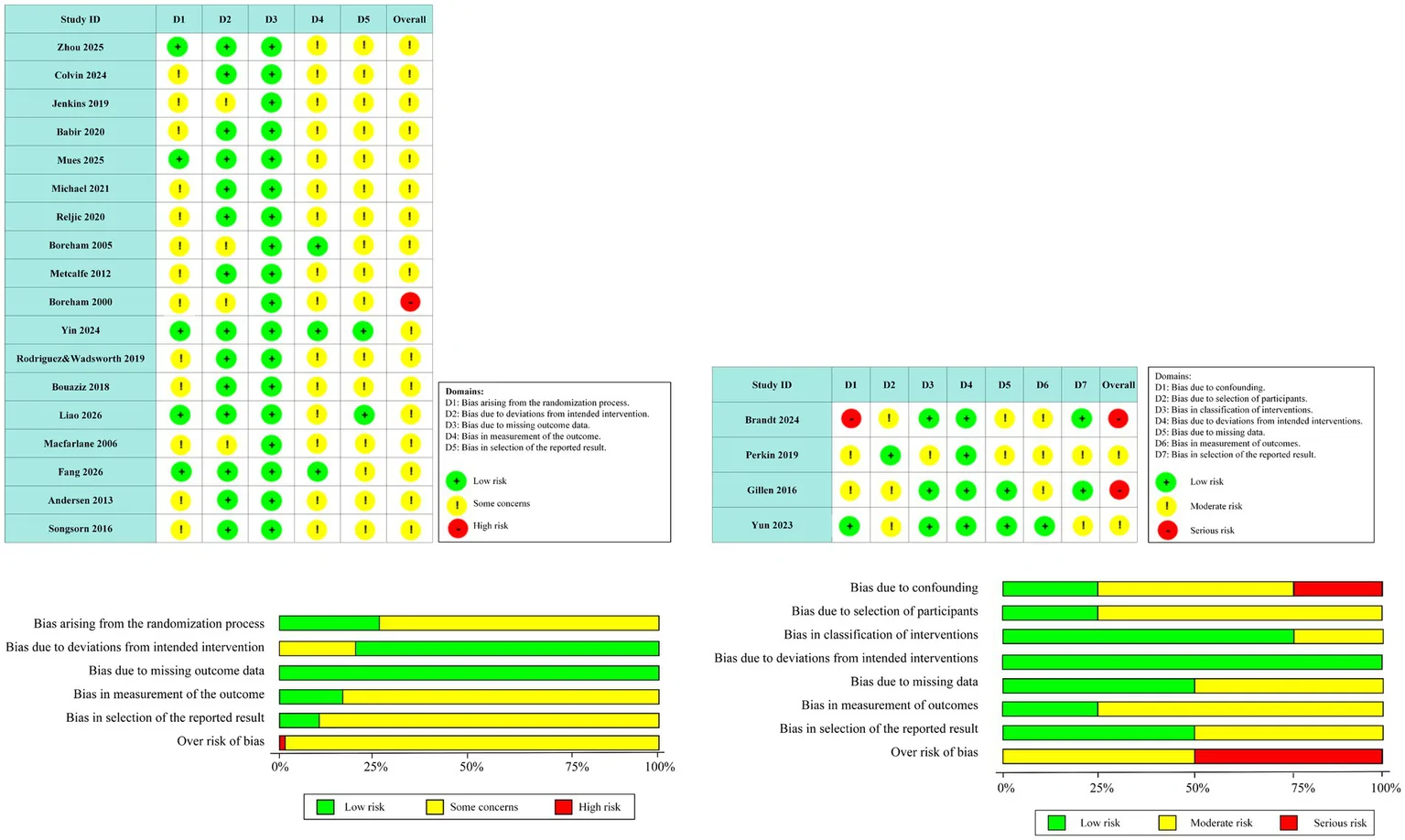
Risk of bias for the included studies.

### 3.6 Sensitivity analysis

In this study, we conducted a sensitivity analysis of the combined effects on various improvement indicators (VO_2max_, VO_2peak_, Triglycerides, and Blood glucose) by systematically excluding each study (Supplementary Table 2). For VO_2max_, after sequentially excluding each study, it was found that excluding individual studies had an extremely minimal impact on the overall effect estimate, indicating that the meta-analysis has high consistency and reliability. Regarding VO_2peak_, when excluding any study other than Babir et al., the results did not show significant differences, suggesting that this study has an important effect on the overall effect estimate. For Triglycerides, excluding any individual study other than the study by Reljic et al. (45) also failed to maintain significance, indicating that this study plays a prominent role in the results. As for blood glucose, excluding the study by Michael et al. may also lead to non-significant results, further emphasizing the potential impact of this study on the overall effect.

Regarding these issues, we believe they may be due to the small sample size. Although preliminary results suggest that ES has some effect in improving these indicators, the large variability in the data means these results should be interpreted with caution. We suggest increasing the sample size and extending the follow-up period in future studies to further validate this effect and improve the stability and generalizability of the research findings.

### 3.7 Certainty of evidence

According to the GRADE evidence grading system, the certainty of evidence for different outcome indicators in this study varies. Among the following indicators, the certainty of evidence for Body fat and Insulin was rated as “Very Low.” The low certainty of evidence for Body fat is mainly due to a wide confidence interval, high heterogeneity between studies, and significant issues of imprecision and inconsistency. The low evidence quality for Insulin is due to a small sample size, a wide confidence interval, and significant heterogeneity between studies, leading to issues of imprecision and inconsistency. In contrast, the evidence quality for VO_2peak_, Muscle mass, Triglycerides, Blood glucose, and Strength was rated as “Low.” The main reasons for the decrease in evidence strength for these indicators include high heterogeneity between studies and small sample sizes. It is worth noting that the certainty of evidence for VO_2max_ was rated as “Moderate,” with the only reason for downgrading being the risk of bias in some studies. Overall, although the exercise snack-based intervention has shown relatively stable and positive effects on VO_2max_, VO_2peak_, Triglycerides, and Blood glucose, the evidence strength for other outcome indicators is relatively weak, thus the interpretation of these results requires more caution. For the GRADE evaluation process of each outcome indicator and the specific reasons for downgrading, please refer to Supplementary Table 3.

### 3.8 Publication Bias

In this study, we also assessed publication bias to determine the potential impact of selective publication on the study results. Although only the VO_2max_ and Body fat indicators met the requirement of including at least 10 studies, we still assessed publication bias for all indicators to ensure the comprehensiveness of the study. The detailed results are shown in Figure 5. Furthermore, for VO_2max_, we found that there was a certain risk of bias. To address this issue, we employed the funnel plot trimming method to correct for bias, with detailed supplementary results provided in Supplementary Table 4. The application of the trimming method helps evaluate and mitigate the impact of publication bias on effect estimates, thereby improving the reliability of the results. Through these measures, we aim to ensure the stability of the research results and provide more accurate reference for future studies in related fields.

**Figure 5:**
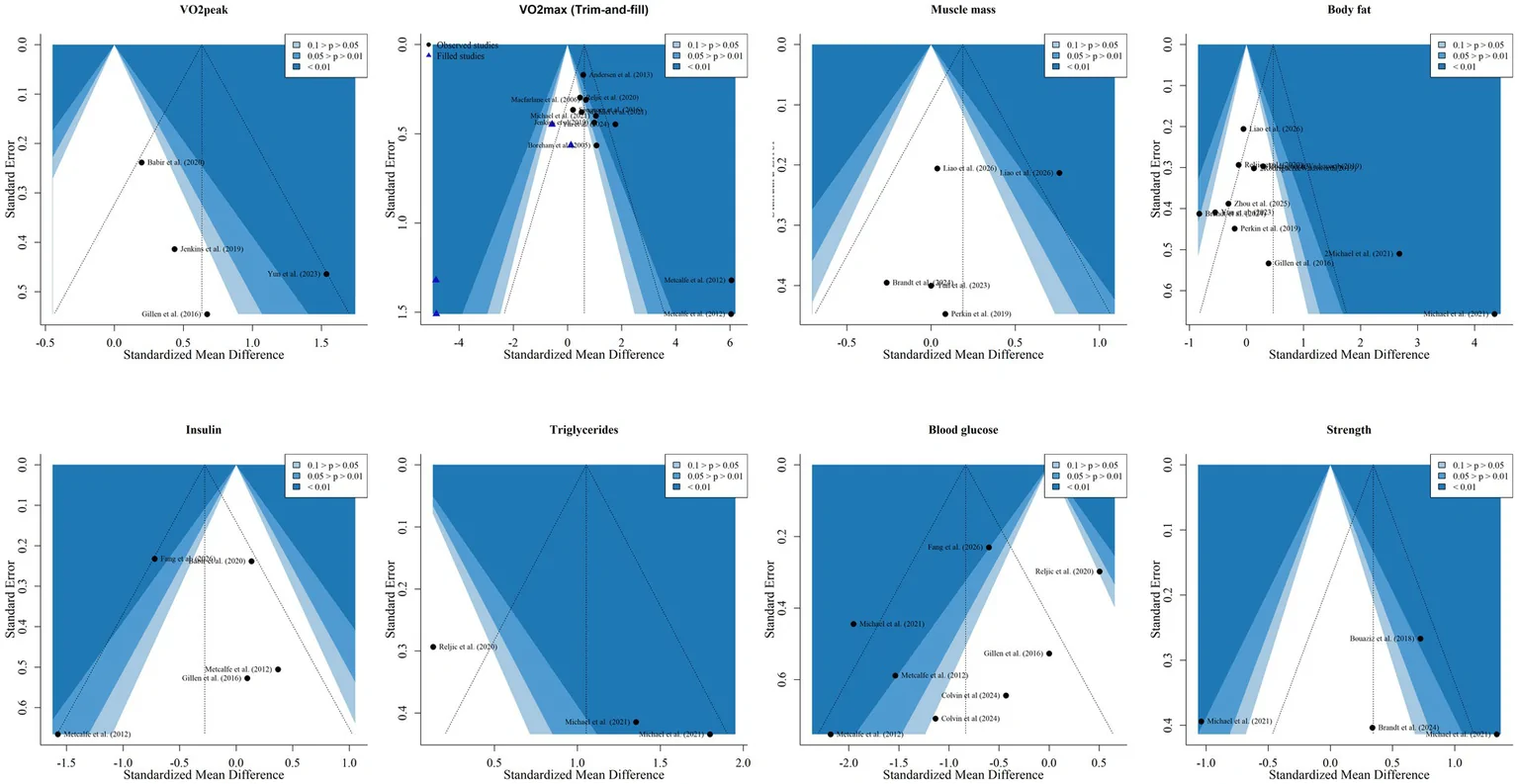
Funnel plot for publication bias detection on outcomes.

### 3.9 Meta-regression

Meta-regression analysis did not find significant moderating effects of age, intervention period, or intervention frequency on the intervention outcomes (Figure 6). Specifically, the relationship between age, intervention period, and intervention frequency and the intervention outcomes did not show statistical significance in the regression analysis (*p*-values > 0.05) (Supplementary Table 5). Therefore, these variables did not effectively predict changes in the intervention outcomes. Since gender is a categorical variable (non-continuous variable), we did not perform a regression analysis on it. The effect of gender is typically assessed through subgroup analysis or other statistical methods suitable for categorical variables. Therefore, the potential impact of gender on intervention outcomes needs to be further explored in future research, particularly under conditions of large sample sizes and multivariable control.

**Figure 6:**
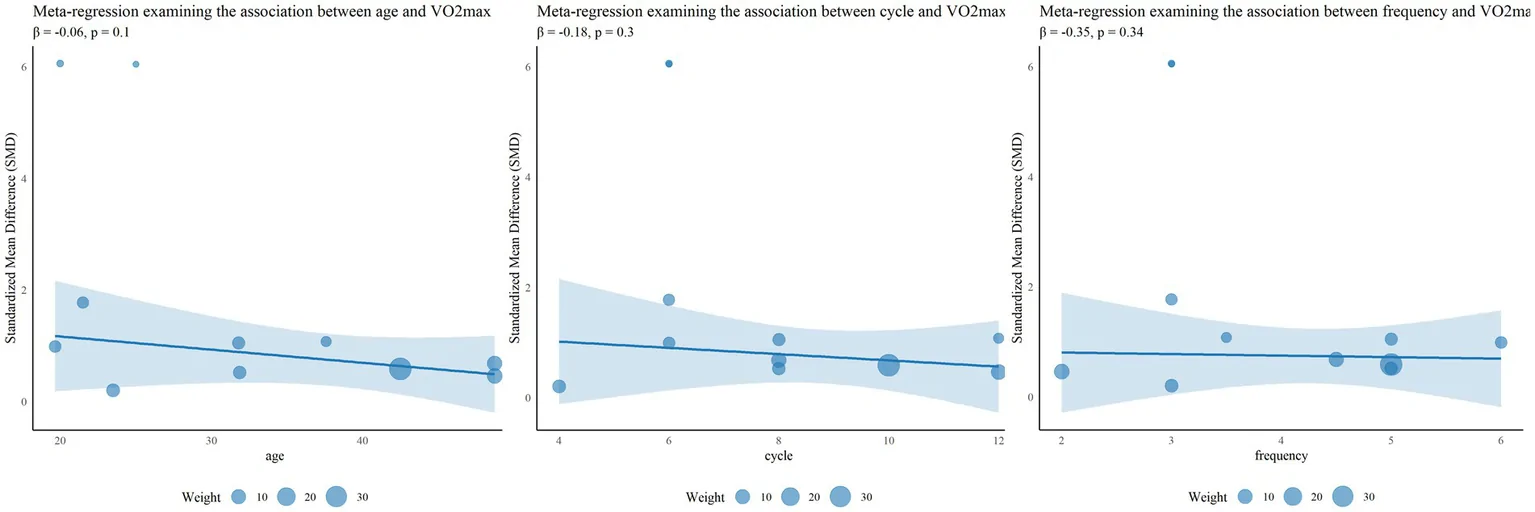
Meta-regression analysis of ES on VO_2max_.

## 4 Discussion

Currently, empirical evidence regarding the effects of ES on sedentary individuals is still limited, especially systematic reviews addressing the comprehensive effects of ES on these populations. This review is the first to explore the effects of ES on cardiorespiratory fitness, body composition, metabolic health, and muscle strength in sedentary individuals. The study not only includes sedentary individuals but also involves individuals with insufficient physical activity, focusing on the effects of ES on these groups with respect to the aforementioned indicators. Our research found that ES significantly improves cardiorespiratory fitness (including VO_2max_ and VO_2peak_) and metabolic health (including triglycerides and blood glucose) in sedentary populations. Although the overall results show strong intervention effects, caution is needed in interpretation, particularly for the VO_2max_ indicator. There is some publication bias in the studies we included, which may affect the robustness of the results. To further investigate these influencing factors, we conducted a subgroup analysis on VO_2max_. Across subgroups stratified by gender, intervention frequency, intervention duration, and age, ES appeared to improve VO_2max_ in sedentary populations. However, substantial heterogeneity remained within several subgroups, with I^2^ values of 82% in the mixed-sex subgroup, 86% in the <5 sessions/week subgroup, 81% in the ≤8 weeks subgroup, and 80% in the ≤44 years subgroup. Meanwhile, no statistically significant differences were observed between subgroups (P for subgroup differences > 0.05), suggesting that the examined subgroup characteristics did not significantly explain the observed heterogeneity. Therefore, although the subgroup analyses suggest a potential beneficial effect of ES on VO_2max_, the substantial residual heterogeneity and potential publication bias warrant cautious interpretation of this finding.

### 4.1 The effect of ES on cardiorespiratory fitness in sedentary individuals

Our meta-analysis results indicate that ES interventions can significantly improve cardiorespiratory fitness in sedentary individuals, with significant improvements in VO_2max_ and VO_2peak_ in the primary analyses. VO_2max_, regarded as the gold standard for measuring cardiopulmonary function (58), reflects the individuals adaptation to exercise load by the heart and lungs, thereby enhancing overall cardiovascular health in sedentary individuals. Improvements in VO_2max_ are primarily achieved by increasing the heart’ s pumping efficiency and enhancing oxygen transport capacity, which contributes to better overall cardiovascular health and increased exercise endurance (59). ES interventions are more frequent and convenient, which enhance the heart’s blood circulation ability while activating the lung’ s gas exchange function. Additionally, exercise promotes mitochondrial biogenesis, improves cellular energy metabolism, and thereby enhances the body’s overall aerobic metabolic capacity (59).

Our findings are consistent with previous related review studies (60), indicating that ES intervention has a positive effect on improving VO_2max_. Additionally, VO_2peak_, as another important indicator of cardiorespiratory fitness, also shows improvement similar to VO_2max_, further proving the comprehensive effect of ES on cardiorespiratory fitness. VO_2peak_ is enhanced under more frequent stimuli, a phenomenon that may be related to the overall improvement in cardiac reserve function, pulmonary function, and oxygen utilization efficiency in peripheral tissues due to exercise (61). ES enhances the body’s ability to absorb and transport oxygen, promoting an increase in blood oxygen saturation, thereby significantly improving VO_2peak_ in the short term. This finding is especially important for sedentary populations, as sedentary behavior has been established as an independent risk factor for cardiovascular diseases, and improving cardiorespiratory fitness is key to reducing these risks (62, 63). Sedentary behavior leads to a decline in the cardiovascular system’s adaptability, manifested as reduced cardiac output and circulatory efficiency (62). Through ES intervention, the cardiorespiratory fitness of sedentary individuals is significantly improved, helping to alleviate these adverse health consequences. Improving cardiorespiratory function not only enhances physical strength but also effectively reduces the incidence of chronic metabolic diseases (64).

However, the finding for VO_2max_ should be interpreted with caution because significant publication bias was detected by Egger’s test (*p* < 0.01). After applying the trim-and-fill method, the adjusted pooled effect for VO_2max_ was reduced from Hedges’ g = 1.24 (95% CI: 0.53 to 1.95, *p* < 0.001) to Hedges’ g = 0.62 (95% CI: −0.59 to 1.83, *p* = 0.32), and the adjusted estimate was no longer statistically significant. This finding suggests that the magnitude of the observed VO_2max_ effect may have been influenced by potential publication bias, and therefore the apparent beneficial effect should not be interpreted as conclusive. Although these results do not necessarily negate a potential benefit of ES, they indicate that the current evidence is insufficient to establish a robust effect of ES on VO_2max_.

Although these improvements are statistically significant in the overall sample, it is important to note that the studies on VO_2max_ in this analysis exhibit high heterogeneity, which may be related to the health status of the participants included in the studies and the limited research evidence available. Therefore, although ES intervention shows significant positive effects on cardiorespiratory fitness, we must interpret these results cautiously and further explore the impact of different intervention variables in future research to optimize intervention strategies and enhance their generalizability.

### 4.2 The effect of ES on body composition in sedentary individuals

The results of the current meta-analysis indicate that ES interventions did not significantly improve the body composition of sedentary individuals, particularly body fat and muscle mass. The ES intervention involves short-duration, intermittent exercises, which may be insufficient to improve body composition in the short term. Changes in body fat and muscle mass typically require longer durations and higher-intensity training (65), but ES interventions failed to provide sufficient intensity and continuity to effectively promote these changes. From a mechanistic perspective, reductions in body fat generally require high energy expenditure, typically achieved through a combination of moderate-to-high-intensity aerobic exercise and strength training (66). Low-to-moderate intensity ES exercise may not significantly increase energy expenditure or promote fat oxidation, especially in sedentary populations, which have weaker metabolic adaptability. Therefore, longer durations and higher-intensity interventions may be necessary. Regarding improvements in muscle mass, although ES may have some effect by increasing activity volume and frequency, its intensity is insufficient to stimulate the strength training stimuli required for muscle synthesis. Significant improvements in muscle mass typically depend on high-intensity strength training, whereas low-intensity aerobic exercise has limited effects on muscle mass improvement, especially in the early stages (65). Additionally, a sedentary lifestyle leads to muscle atrophy and decreased metabolic rate (67). While ES can provide exercise intervention, it may not be sufficient to counteract the negative effects of a sedentary lifestyle. Improving body composition in sedentary individuals requires a combination of strength training and a more comprehensive exercise regimen. Since the maximum intervention period in the included studies is 12 weeks, future research could extend the intervention period to explore the long-term effects of ES on body composition.

### 4.3 The effect of ES on metabolic health in sedentary individuals

In terms of metabolic health, this study primarily assessed three key indicators: blood glucose, insulin, and triglycerides. The results indicated that ES intervention significantly improved blood glucose and triglycerides, but did not significantly improve insulin levels. This finding suggests that ES intervention can effectively improve certain metabolic health indicators, particularly those related to fat metabolism and blood glucose control, but its impact on insulin is relatively weak. The improvement in blood glucose and triglycerides may be related to the increase in metabolic rate and the promotion of fat oxidation by ES (68). Short-duration, high-frequency exercise can promote glucose uptake and metabolism, thereby improving blood glucose control (69). Furthermore, ES helps reduce fat storage by increasing exercise frequency, which is closely related to the reduction in triglyceride levels. Studies have shown that regular aerobic exercise can reduce triglyceride levels in the blood by regulating fatty acid metabolism, improve lipid profiles, and thereby lower the risk of cardiovascular diseases (70). However, the improvement in insulin levels due to ES is relatively weak, possibly because improving insulin resistance typically requires longer durations and higher-intensity interventions (71). In conclusion, ES has shown positive effects on improving blood glucose and triglycerides, but its impact on insulin should be interpreted with caution. Future research could consider increasing the intensity and duration of the intervention, exploring the potential of combining ES with other high-intensity training methods to further improve insulin sensitivity, and thus more effectively enhance the metabolic health of sedentary individuals.

### 4.4 The effect of ES on muscle strength in sedentary individuals

The results of this meta-analysis indicate that ES intervention did not significantly improve muscle strength in sedentary individuals. Although ES increased physical activity through short-duration, intermittent exercise forms, its relatively short intervention time may have limited its effects on muscle strength improvement. Previous studies have shown that traditional resistance training, particularly higher-load training, is generally more effective in promoting muscle strength improvements, accompanied by enhancements in muscle morphology and neural adaptations (72, 73). In contrast, the short-duration, intermittent nature of ES activities may not provide sufficient and continuous mechanical stimuli, thus making it difficult to effectively induce protein synthesis and structural adaptations related to muscle strength growth (74).

### 4.5 Considerations on the application of ES in sedentary individuals

Sedentary individuals include college students, office workers, physically inactive elderly individuals, and other special groups. They often struggle to sustain regular exercise interventions due to a lack of interest in physical activity, time constraints, or limitations imposed by their work style. Taking office workers as an example, prolonged desk work often makes it difficult for them to reach the recommended levels of physical activity; similarly, professionals with a fast-paced work environment and heavy task burdens, such as healthcare workers, face real challenges in engaging in regular physical exercise (75). In this context, ES, as a form of exercise that is short in duration and flexible in format, can provide sustainable health benefits to these populations without significantly affecting their daily work and life schedules. For example, healthcare workers or other busy professionals can choose to walk up and down stairs instead of using the elevator to increase their daily physical activity; office workers can take advantage of work breaks to perform simple bodyweight exercises or use portable exercise equipment for brief workouts, thus achieving moderate exercise during their busy work schedules. Although the duration of each ES session is relatively short, it can effectively help sedentary populations increase their overall physical activity and promote health improvements without significantly affecting work efficiency. Therefore, ES interventions, as a low-barrier and easily implementable form of exercise, have significant practical application potential, especially in improving the overall health of sedentary populations. In conclusion, the unique advantage of ES lies in its ability to integrate well into daily life scenarios, especially for those who find it difficult to engage in regular or high-intensity exercise due to the nature of their work or lifestyle constraints. Future research could further explore the specific forms, applicable conditions, and optimization strategies of ES interventions, thereby providing more individualized and actionable health promotion plans for different populations.

### 4.6 Limitations

Currently, research on the application of ES interventions in sedentary individuals is still limited, and there are significant differences in the types of interventions and outcome measures, which affects the comparability and interpretability of the results. Moreover, the implementation standards for ES are still unclear, leading to considerable variation in the intervention methods and execution standards in the studies. In practical applications, many sedentary individuals, particularly office workers, may find it difficult to adhere to strict ES intervention protocols due to prolonged psychological stress and environmental constraints, which can affect the effectiveness and sustainability of the intervention. Additionally, most of the outcome measures included in this study are supported by a limited number of studies, which did not meet the requirements for further subgroup analyses and funnel plots, potentially impacting the robustness and generalizability of the findings. Furthermore, the review was registered in PROSPERO on March 21, 2026, after completion of the literature search on February 5, 2026. Although the review protocol and methodological framework had been established before the review was conducted, the PROSPERO registration was completed after the search due to the registration and administrative process. We acknowledge that the retrospective timing of registration represents a limitation and may reduce the extent to which prospective registration can safeguard against potential *post-hoc* methodological changes. Nevertheless, this study provides preliminary evidence for the application of ES in sedentary populations, but it still requires validation under more rigorous experimental conditions. Future research should enhance the control of intervention variables, particularly the effects of ES interventions under different population and environmental conditions, to more clearly define the impact of different types of ES interventions on sedentary individuals and provide theoretical foundations for developing suitable exercise programs.

## 5 Conclusion

This study preliminarily integrated the effective improvements of ES interventions on cardiorespiratory fitness and certain aspects of metabolic health in sedentary populations, showing significant positive effects, particularly on VO_2peak_, triglycerides, and blood glucose, while the apparent benefit for VO_2max_ was not maintained after trim-and-fill adjustment. However, ES interventions did not significantly improve body composition and muscle strength in sedentary individuals. These findings suggest that ES interventions have potential in improving cardiorespiratory and metabolic health, but their impact on body composition and muscle strength remains limited. However, given that the evidence quality for most outcomes is low, these results should be interpreted with caution. Future research should further explore the long-term effects of ES, particularly through follow-up studies to validate its sustainability and long-term benefits. At the same time, intervention protocols should be optimized, taking into account the needs of different populations, intervention intensity, and frequency, in order to provide more effective and sustainable exercise intervention strategies for sedentary individuals. Furthermore, further research should focus on the potential effect of different types of ES interventions on body composition and muscle strength, providing stronger evidence for developing more comprehensive exercise programs.

## Data Availability

The original contributions presented in the study are included in the article/Supplementary material, further inquiries can be directed to the corresponding author.

## Footnotes

1
https://apps.automeris.io/wpd/
