# Supplementary material for: The effect of exercise snacks on cardiorespiratory fitness, body composition, metabolic health, and muscle strength in sedentary individuals: a systematic review and meta-analysis
Source: Front Nutr. 2026 Sep 28;13:1883923. doi: 10.3389/fnut.2026.1883923 (PMC13654994; doi:10.3389/fnut.2026.1883923)
Supplement: Supplementary file 1 [file Table_1.docx]

**Supplementary Information**

**The Effect of Exercise Snacks on Cardiorespiratory Fitness, Body Composition, Metabolic Health, and Muscle Strength in Sedentary Individuals: A Systematic Review and Meta-Analysis**

Kai Qi^1,2†^, Qiyi Wang^1,3†^, Decheng Xu^1,3^, Adam Kawczyński^4^, Aiguo Chen^1*^

^1^School of Sport and Brain Health, Nanjing Sport Institute, Nanjing, China

^2^Gdansk University of Physical Education and Sport, Gdansk, Poland

^3^College of Physical Education, Yangzhou University, Yangzhou, China

^4^Faculty of Medicine, Wrocław University of Science and Technology, Wrocław, Poland

^†^These authors contributed substantially the same amount to this work and should be considered co-first authors.

^*^ **Correspondence:** Aiguo Chen (agchen@nsi.edu.cn)

| **Section and Topic** | **Item #** | **Checklist item** | **Location where item is reported** |
| --- | --- | --- | --- |
| **TITLE** | | |  |
| Title | 1 | Identify the report as a systematic review. | Pages 1 |
| **ABSTRACT** | | |  |
| Abstract | 2 | See the PRISMA 2020 for Abstracts checklist. | Pages 1 |
| **INTRODUCTION** | | |  |
| Rationale | 3 | Describe the rationale for the review in the context of existing knowledge. | Pages 2 |
| Objectives | 4 | Provide an explicit statement of the objective(s) or question(s) the review addresses. | Pages 2 |
| **METHODS** | | |  |
| Eligibility criteria | 5 | Specify the inclusion and exclusion criteria for the review and how studies were grouped for the syntheses. | Pages 3 |
| Information sources | 6 | Specify all databases, registers, websites, organisations, reference lists and other sources searched or consulted to identify studies. Specify the date when each source was last searched or consulted. | Pages 2 |
| Search strategy | 7 | Present the full search strategies for all databases, registers and websites, including any filters and limits used. | Pages 2-3 |
| Selection process | 8 | Specify the methods used to decide whether a study met the inclusion criteria of the review, including how many reviewers screened each record and each report retrieved, whether they worked independently, and if applicable, details of automation tools used in the process. | Pages 3 |
| Data collection process | 9 | Specify the methods used to collect data from reports, including how many reviewers collected data from each report, whether they worked independently, any processes for obtaining or confirming data from study investigators, and if applicable, details of automation tools used in the process. | Pages 3-4 |
| Data items | 10a | List and define all outcomes for which data were sought. Specify whether all results that were compatible with each outcome domain in each study were sought (e.g. for all measures, time points, analyses), and if not, the methods used to decide which results to collect. | Pages 4 |
|  | 10b | List and define all other variables for which data were sought (e.g. participant and intervention characteristics, funding sources). Describe any assumptions made about any missing or unclear information. | Pages 4 |
| Study risk of bias assessment | 11 | Specify the methods used to assess risk of bias in the included studies, including details of the tool(s) used, how many reviewers assessed each study and whether they worked independently, and if applicable, details of automation tools used in the process. | Pages 4 |
| Effect measures | 12 | Specify for each outcome the effect measure(s) (e.g. risk ratio, mean difference) used in the synthesis or presentation of results. | Pages 4 |
| Synthesis methods | 13a | Describe the processes used to decide which studies were eligible for each synthesis (e.g. tabulating the study intervention characteristics and comparing against the planned groups for each synthesis (item #5)). | Pages 4 |
|  | 13b | Describe any methods required to prepare the data for presentation or synthesis, such as handling of missing summary statistics, or data conversions. | Pages 4 |
|  | 13c | Describe any methods used to tabulate or visually display results of individual studies and syntheses. | Pages 4 |
|  | 13d | Describe any methods used to synthesize results and provide a rationale for the choice(s). If meta-analysis was performed, describe the model(s), method(s) to identify the presence and extent of statistical heterogeneity, and software package(s) used. | Pages 4 |
|  | 13e | Describe any methods used to explore possible causes of heterogeneity among study results (e.g. subgroup analysis, meta-regression). | Pages 4 |
|  | 13f | Describe any sensitivity analyses conducted to assess robustness of the synthesized results. | Pages 4 |
| Reporting bias assessment | 14 | Describe any methods used to assess risk of bias due to missing results in a synthesis (arising from reporting biases). | Pages 4 |
| Certainty assessment | 15 | Describe any methods used to assess certainty (or confidence) in the body of evidence for an outcome. | Pages 4 |
| **RESULTS** | | |  |
| Study selection | 16a | Describe the results of the search and selection process, from the number of records identified in the search to the number of studies included in the review, ideally using a flow diagram. | Pages 4 |
|  | 16b | Cite studies that might appear to meet the inclusion criteria, but which were excluded, and explain why they were excluded. | Pages 4 |
| Study characteristics | 17 | Cite each included study and present its characteristics. | Pages 4-11 |
| Risk of bias in studies | 18 | Present assessments of risk of bias for each included study. | Pages 12-13 |
| Results of individual studies | 19 | For all outcomes, present, for each study: (a) summary statistics for each group (where appropriate) and (b) an effect estimate and its precision (e.g. confidence/credible interval), ideally using structured tables or plots. | Pages 11-13 |
| Results of syntheses | 20a | For each synthesis, briefly summarise the characteristics and risk of bias among contributing studies. | Pages 11-13 |
|  | 20b | Present results of all statistical syntheses conducted. If meta-analysis was done, present for each the summary estimate and its precision (e.g. confidence/credible interval) and measures of statistical heterogeneity. If comparing groups, describe the direction of the effect. | Pages 11-13 |
|  | 20c | Present results of all investigations of possible causes of heterogeneity among study results. | Pages 14 |
|  | 20d | Present results of all sensitivity analyses conducted to assess the robustness of the synthesized results. | Pages 14 |
| Reporting biases | 21 | Present assessments of risk of bias due to missing results (arising from reporting biases) for each synthesis assessed. | Pages 14-15 |
| Certainty of evidence | 22 | Present assessments of certainty (or confidence) in the body of evidence for each outcome assessed. | Pages 14 |
| **DISCUSSION** | | |  |
| Discussion | 23a | Provide a general interpretation of the results in the context of other evidence. | Pages 15-17 |
|  | 23b | Discuss any limitations of the evidence included in the review. | Pages 17 |
|  | 23c | Discuss any limitations of the review processes used. | Pages 17 |
|  | 23d | Discuss implications of the results for practice, policy, and future research. | Pages 17 |
| **OTHER INFORMATION** | | |  |
| Registration and protocol | 24a | Provide registration information for the review, including register name and registration number, or state that the review was not registered. | Pages 2 |
|  | 24b | Indicate where the review protocol can be accessed, or state that a protocol was not prepared. | Not Applicable |
|  | 24c | Describe and explain any amendments to information provided at registration or in the protocol. | Not Applicable |
| Support | 25 | Describe sources of financial or non-financial support for the review, and the role of the funders or sponsors in the review. | Pages 18 |
| Competing interests | 26 | Declare any competing interests of review authors. | Pages 18 |
| Availability of data, code and other materials | 27 | Report which of the following are publicly available and where they can be found: template data collection forms; data extracted from included studies; data used for all analyses; analytic code; any other materials used in the review. | Pages 17-18 |

*From:* Page MJ, McKenzie JE, Bossuyt PM, Boutron I, Hoffmann TC, Mulrow CD, et al. The PRISMA 2020 statement: an updated guideline for reporting systematic reviews. BMJ 2021;372:n71. doi: 10.1136/bmj.n71. This work is licensed under CC BY 4.0. To view a copy of this license, visit <https://creativecommons.org/licenses/by/4.0/>

**Search strategy**

| Data | Query | Results |
| --- | --- | --- |
| PubMed | (((((((((Exercise snack[Title/Abstract]) OR (Snacktivity[Title/Abstract])) OR (Short bouts of exercise[Title/Abstract])) OR (Physical activity short bouts[Title/Abstract])) OR (Exercise short bouts[Title/Abstract])) OR (Short bouts of stair climbing[Title/Abstract])) AND (sedentary[Title/Abstract])) OR (sedentary population[Title/Abstract])) OR (sedentary individuals[Title/Abstract])) OR (physically inactive population[Title/Abstract]) | 1378 |
| Web of Science | (((((((((TS=(Exercise snack)) OR TS=(sractivity)) OR TS=(Short bouts of exercise)) OR TS=(Physical activity short bouts)) OR TS=(Exercise short bouts)) OR TS=(Short bouts of stair climbing)) AND TS=(sedentary)) OR TS=(sedentary population)) OR TS=(sedentary individuals)) OR TS=(physically inactive population) | 6913 |
| SciELO | (((((((((TS=(Exercise snack)) OR TS=(sractivity)) OR TS=(Short bouts of exercise)) OR TS=(Physical activity short bouts)) OR TS=(Exercise short bouts)) OR TS=(Short bouts of stair climbing)) AND TS=(sedentary)) OR TS=(sedentary population)) OR TS=(sedentary individuals)) OR TS=(physically inactive population) | 1078 |
| Cochrane Central Register of Controlled Trials (CENTRAL) | MeSH descriptor: [Sedentary Behavior] explode all trees or (sedentary population or sedentary individuals or physically inactive population):ti,ab,kw (Word variations have been searched) and (Exercise snack or Snacktivity or Short bouts of exercise or Physical activity short bouts or Exercise short bouts or Short bouts of stair climbing):ti,ab,kw (Word variations have been searched) | 156 |
| CNKI | （主题：运动零食）OR（主题：运动）AND（主题：久坐）OR（主题： 久坐人群） | 456 |

Supplementary Table 1. Subgroup analyses based on VO_2max_.

| **Subgroup** | **k** | **SMD** | **95%CI** | **p** | **I²** | **p for SA** |  |
| --- | --- | --- | --- | --- | --- | --- | --- |
| Sex |  |  |  |  |  | 0.23 |  |
| Female | 3 | 0.83 | [0.34, 1.31] | 0.001 | 0.00 |  |  |
| Mixed | 8 | 1.73 | [0.34, 3.11] | 0.014 | 0.82 |  |  |
| Cycle |  |  |  |  |  | 0.14 |  |
| <5 | 6 | 2.22 | [0.21, 4.23] | 0.031 | 0.86 |  |  |
| ≥5 | 5 | 0.69 | [0.43, 0.95] | 0 | 0 |  |  |
| Frequency |  |  |  |  |  | 0.09 |  |
| >8 weeks | 3 | 0.59 | [0.31, 0.87] | 0 | 0 |  |  |
| ≤8 weeks | 8 | 1.79 | [0.44, 3.14] | 0.009 | 0.81 |  |  |
| Age |  |  |  |  |  | 0.07 |  |
| ≤44 | 8 | 1.84 | [0.51, 3.18] | 0.007 | 0.8 |  |  |
| 45-59 | 3 | 0.58 | [0.31, 0.84] | 0 | 0 |  |  |
| Note. SA = subgroup analysis | | | | | | | |

Supplementary Table 2. Results of sensitivity analysis.

| **Omitted study** | **Hedges’ g** | **95%CI** | **p** |
| --- | --- | --- | --- |
| **Cardiorespiratory Fitness** |  |  |  |
| **VO_2max_** |  |  |  |
| 1Omitting Michael et al. (2021) | 1.44 | (0.52, 2.35) | <0.01 |
| 2Omitting Michael et al. (2021) | 1.4 | (0.45, 2.34) | <0.01 |
| Omitting Reljic et al. (2020) | 1.44 | (0.53, 2.36) | <0.01 |
| Omitting Boreham et al. (2005) | 1.39 | (0.45, 2.32) | <0.01 |
| Omitting Yin et al. (2024) | 0.68 | (0.47, 0.89) | <0.001 |
| Omitting Macfarlane et al. (2006) | 1.43 | (0.50, 2.37) | <0.01 |
| Omitting Andersen et al. (2013) | 1.44 | (0.51, 2.37) | <0.01 |
| 1Omitting Metcalfe et al. (2012) | 0.81 | (0.50, 1.12) | <0.001 |
| 2Omitting Metcalfe et al. (2012) | 0.76 | (0.50, 1.03) | <0.001 |
| Omitting Songsorn et al. (2016) | 1.44 | (0.57, 2.31) | <0.01 |
| Omitting Jenkins et al(2019) | 1.4 | (0.46, 2.35) | <0.01 |
| **VO_2peak_** |  |  |  |
| Omitting Jenkins et al. (2019) | 0.73 | (-0.08, 1.55) | 0.08 |
| Omitting Babir et al. (2020) | 0.87 | (0.18, 1.56) | <0.05 |
| Omitting Gillen et al. (2016) | 0.65 | (-0.12, 1.42) | 0.1 |
| Omitting Yun et al. (2023) | 0.31 | (-0.07, 0.69) | 0.11 |
| **Body Composition** |  |  |  |
| **Muscle mass** |  |  |  |
| Omitting Brandt et al. (2024) | 0.27 | (-0.16, 0.70) | 0.21 |
| Omitting Perkin et al. (2019) | 0.19 | (-0.27, 0.66) | 0.41 |
| 1Omitting Liao et al. (2026) | 0.23 | (-0.29, 0.74) | 0.39 |
| 2Omitting Liao et al. (2026) | -0.01 | (-0.31, 0.30) | 0.95 |
| Omitting Yun et al. (2023) | 0.21 | (-0.26, 0.68) | 0.37 |
| **Body fat** |  |  |  |
| Omitting Zhou et al. (2025) | 0.55 | (-0.39, 1.50) | 0.25 |
| Omitting Brandt et al. (2024) | 0.6 | (-0.31, 1.51) | 0.2 |
| Omitting Perkin et al. (2019) | 0.54 | (-0.41, 1.49) | 0.26 |
| Omitting Gillen et al. (2016) | 0.48 | (-0.47, 1.44) | 0.32 |
| 1Omitting Michael et al. (2021) | 0.1 | (-0.43, 0.64) | 0.7 |
| 2Omitting Michael et al. (2021) | 0.25 | (-0.56, 1.05) | 0.55 |
| Omitting Reljic et al. (2020) | 0.54 | (-0.42, 1.49) | 0.27 |
| 1Omitting Rodriguez & Wadsworth (2019) | 0.49 | (-0.47, 1.45) | 0.32 |
| 2Omitting Rodriguez & Wadsworth (2019) | 0.51 | (-0.45, 1.47) | 0.3 |
| Omitting Liao et al. (2026) | 0.53 | (-0.43, 1.49) | 0.28 |
| Omitting Yun et al. (2023) | 0.57 | (-0.36, 1.50) | 0.23 |
| **Metabolic Health** |  |  |  |
| **Insulin** |  |  |  |
| Omitting Babir et al. (2020) | -0.43 | (-1.17, 0.31) | 0.25 |
| Omitting Gillen et al. (2016) | -0.36 | (-1.09, 0.36) | 0.33 |
| 1Omitting Metcalfe et al. (2012) | -0.11 | (-0.65, 0.43) | 0.69 |
| 2Omitting Metcalfe et al. (2012) | -0.41 | (-1.08, 0.25) | 0.22 |
| Omitting Fang et al. (2026) | -0.1 | (-0.77, 0.56) | 0.76 |
| **Triglycerides** | |  |  |
| 1Omitting Michael et al. (2021) | 0.71 | (-0.49, 1.90) | 0.25 |
| 2Omitting Michael et al. (2021) | 0.93 | (-0.70, 2.57) | 0.26 |
| Omitting Reljic et al. (2020) | 1.57 | (0.98, 2.15) | <0.001 |
| **Blood glucose** |  |  |  |
| 1Omitting Colvin et al. (2024) | -0.81 | (-1.56, -0.05) | <0.05 |
| 2Omitting Colvin et al. (2024) | -0.89 | (-1.66, -0.13) | <0.05 |
| Omitting Gillen et al. (2016) | -0.96 | (-1.70, -0.21) | <0.05 |
| Omitting Michael et al. (2021) | -0.64 | (-1.30, 0.03) | 0.06 |
| Omitting Reljic et al. (2020) | -1.06 | (-1.66, -0.46) | <0.001 |
| 1Omitting Metcalfe et al. (2012) | -0.69 | (-1.37, -0.00) | <0.05 |
| 2Omitting Metcalfe et al. (2012) | -0.75 | (-1.49, -0.00) | <0.05 |
| Omitting Fang et al. (2026) | -0.89 | (-1.70, -0.09) | <0.05 |
| **Muscle Strength** |  |  |  |
| **Strength** |  |  |  |
| Omitting Brandt et al. (2024) | 0.34 | (-1.03, 1.72) | 0.62 |
| 1Omitting Michael et al. (2021) | 0.78 | (0.32, 1.24) | <0.001 |
| 2Omitting Michael et al. (2021) | 0.03 | (-1.02, 1.08) | 0.95 |
| Omitting Bouaziz et al. (2018) | 0.21 | (-1.14, 1.56) | 0.76 |

Note: 1Omitting Michael et al. (2021)-Home-Based Stair Climbing; 2Omitting Michael et al. (2021)-Gym-Based Stair Climbing; 1Omitting Metcalfe et al. (2012) and 2Omitting Metcalfe et al. (2012)-Reduced-Exertion High-intensity interval training; 1Omitting Liao et al. (2026) and 2Omitting Liao et al. (2026)-high-intensity circuit training; 1Omitting Rodriguez & Wadsworth (2019)-multiple bouts of walking; 2Omitting Rodriguez & Wadsworth (2019)-continuous walking; 1Omitting Colvin et al. (2024)-Yoga; 2Omitting Colvin et al. (2024)-Tai Chi.

Supplementary Table 3. GRADE Evidence Level Results

| **Outcome** | **Participant** | **Level of evidence** | | | | | **Hedges’g (95%CI)** | **Certainty of evidence** |
| --- | --- | --- | --- | --- | --- | --- | --- | --- |
|  |  | **Risk of bias** | **Inconsistency** | **Indirectness** | **Imprecision** | **Publicationbias** |  |  |
| VO_2max_ | 451  (11 RCTs) | Serious | Not serious | Not serious | Not serious | Serious | 1.24 (0.53, 1.95) | Moderate |
| VO_2peak_ | 125  (4 RCTs) | Not serious | Serious | Not serious | Serious | Not serious | 0.63 (0.05, 1.22) | Low |
| Muscle mass | 263  (5 RCTs) | Not serious | Not serious | Not serious | Serious | Not serious | 0.19 (-0.21, 0.59) | Low |
| Body fat | 418  (11 RCTs) | Serious | Serious | Not serious | Not serious | Serious | 0.47 (-0.39, 1.33) | Very low |
| Insulin | 194  (5 RCTs) | Serious | Serious | Not serious | Serious | Not serious | -0.28 (-0.86, 0.30) | Very low |
| Triglycerides | 119  (3 RCTs) | Not serious | Serious | Not serious | Serious | Not serious | 1.05 (0.05, 2.06) | Low |
| Blood glucose | 228  (8 RCTs) | Not serious | Serious | Not serious | Serious | Not serious | -0.83 (-1.51, -0.15) | Low |
| Strength | 155  (4 RCTs) | Not serious | Serious | Not serious | Serious | Not serious | 0.34 (-0.62, 1.31) | Low |

GRADE quality of clinical evidence and grade of recommendation:

High: The group has full confidence in the results

Medium: Subjects are moderately confident of the outcome

Low: Subjects have limited confidence in the results

Very low: subject group has very limited confidence in the results

Supplementary Table 4. The result of publication bias in outcome indicators after applying the trimming method

| Variables | *k* | Egger’ s test  p-value | Publication bias | Effect size and 95% CI after applying the trimming method |
| --- | --- | --- | --- | --- |
| Insulin | 5 |  | NA (<10 studies) | NA |
| Muscle mass | 5 |  | NA (<10 studies) | NA |
| Strength | 4 |  | NA (<10 studies) | NA |
| Triglycerides | 3 |  | NA (<10 studies) | NA |
| VO_2max_ | 11 | <0.01 | Yes | 0.62 (-0.59, 1.83), p = 0.32 |
| VO_2peak_ | 4 |  | NA (<10 studies) | NA |
| Body fat | 11 | 0.12 | No | NA |
| Blood glucose | 8 |  | NA (<10 studies) | NA |
| Note. NA = Not applicable | | | | |

Supplementary Table 5. Meta-regression analysis of ES on VO_2max_

| Moderator | β | SE | p | 95%CI | | τ² |
| --- | --- | --- | --- | --- | --- | --- |
|  |  |  |  | lower | upper |  |
| Cycle | -0.18 | 0.17 | 0.30 | -0.52 | 0.16 | 1.74 |
| Frequency | -0.35 | 0.37 | 0.34 | -1.08 | 0.37 | 1.85 |
| Age | -0.06 | 0.04 | 0.10 | -0.13 | 0.01 | 1.25 |
